# Protective Unfolded Protein Response in Human Pancreatic Beta Cells Transplanted into Mice

**DOI:** 10.1371/journal.pone.0011211

**Published:** 2010-06-18

**Authors:** Jeffrey Kennedy, Hitoshi Katsuta, Min-Ho Jung, Lorella Marselli, Allison B. Goldfine, Ulysses J. Balis, Dennis Sgroi, Susan Bonner-Weir, Gordon C. Weir

**Affiliations:** 1 Section on Islet Cell Biology and Regenerative Medicine, Research Division, Joslin Diabetes Center, and the Department of Medicine, Harvard Medical School, Boston, Massachusetts, United States of America; 2 Molecular Pathology Unit, Massachusetts General Hospital, Harvard Medical School, Boston, Massachusetts, United States of America; University of Bremen, Germany

## Abstract

**Background:**

There is great interest about the possible contribution of ER stress to the apoptosis of pancreatic beta cells in the diabetic state and with islet transplantation.

**Methods and Findings:**

Expression of genes involved in ER stress were examined in beta cell enriched tissue obtained with laser capture microdissection (LCM) from frozen sections of pancreases obtained from non-diabetic subjects at surgery and from human islets transplanted into ICR-SCID mice for 4 wk. Because mice have higher glucose levels than humans, the transplanted beta cells were exposed to mild hyperglycemia and the abnormal environment of the transplant site. RNA was extracted from the LCM specimens, amplified and then subjected to microarray analysis. The transplanted beta cells showed an unfolded protein response (UPR). There was activation of many genes of the IRE-1 pathway that provide protection against the deleterious effects of ER stress, increased expression of ER chaperones and ERAD (ER-associated protein degradation) proteins. The other two arms of ER stress, PERK and ATF-6, had many down regulated genes. Downregulation of EIF2A could protect by inhibiting protein synthesis. Two genes known to contribute to apoptosis, CHOP and JNK, were downregulated.

**Conclusions:**

Human beta cells in a transplant site had UPR changes in gene expression that protect against the proapoptotic effects of unfolded proteins.

## Introduction

Over the past 20 years the proof-of-principle of reversing the diabetic state with islet transplantation has been established, however, serious limitations remain [1]. Among other problems, the grafts typically lose their efficacy within months to a few years, they usually provide less than perfect glucose control, and the availability of healthy islets for these transplants is very limited. There are indications that insulin secretion is impaired in a transplant site compared to that from beta cells in their native environment [2]. There seems to be little capacity for regeneration and there is a presumed increase in the rate of apoptosis. These problems can be partially attributed to the effects of host immune rejection, autoimmunity, and immunosuppressive drugs. However, glucose toxicity [3] and the abnormal transplant environment [4] also are likely to contribute to the altered functional capacity of transplanted beta cells. Similar processes have been implicated in the pathogenesis of type 2 diabetes [5], [6].

The endoplasmic reticulum (ER) stress response can protect cells when the cell's unfolded protein load exceeds the endoplasmic reticulum's folding capacity. ER stress responses include induction of ER chaperone proteins, translational attenuation, ER associated protein degradation, and when ER stress is severe, apoptosis [7]. ER stress responses have been linked to beta cell failure in type 2 diabetes [8], [9], [10], [11], [12], [13], but most of the supportive studies have been performed with *in vitro* measurements.

The ER stress response is regulated by levels of BiP (heat shock 70kDa protein 5, HSPA5, GRP78), an ER chaperone protein, free in the endoplasmic reticulum. When the ER folding load is increased, free BiP levels decrease and three ER stress pathways are initiated:

1. IRE-1 (endoplasmic reticulum to nucleus signaling 1) splices XBP-1 (X box binding protein-1) resulting in translation of transcription factor XBP-1 and increased production of ER chaperones and ERAD (ER-associated protein degradation) proteins [7].
2. PERK (protein kinase-like endoplasmic reticulum kinase) phosphorylates eIF2α (eukaryotic translation initiation factor 2A) resulting in generalized attenuation of translation but stimulated translation of transcription factor ATF-4 (activating transcription factor 4). ATF-4 initiates a cascade promoting transcription of the pro-apoptotic factor CHOP (C/EBP homologous protein, DDIT3) [7], [14].
3. Transcription factor ATF-6 (activating transcription factor 6) promotes transcription of chaperone proteins, pro-apoptotic factor CHOP, ERAD associated proteins, and the PERK inhibitor DNA-JC3 (P58IRK, HSP40) [7], [15].

The IRE-1 pathway results mainly in cell adaptation and increased protein folding capacity, while activation of PERK and ATF-6 pathways lead to increased levels of pro-apoptotic components.

The present study was undertaken to determine if ER stress response mechanisms contribute to the dysfunction of human beta cells in a transplant site exposed to mild hyperglycemia. Thus, mRNA expression of beta cells from tissue obtained from non-diabetic subjects at surgery was compared to that of human beta cells transplanted under the kidney capsule of SCID mice in order to examine the difference in gene expression of beta cells in their native environment compared to the in an abnormal transplant environment, similar to that found with successful clinical transplants [16]. Nearly pure pancreatic beta cell populations were isolated by laser capture microdissection, extracted for RNA and subjected to microarray analysis. The results show an ER stress response in these beta cells but indicate that it is likely serving a protective role rather than a destructive one.

## Methods

### Ethics Statement

Tissue samples were obtained and records reviewed with IRB approval from Partners Healthcare and Joslin Diabetes Center. The study of the discarded human tissue and review of medical records was considered exempt from informed consent by both of these IRBs. The Joslin Animal Care Committee approved all animal experiments (protocol #90-07).

### Surgical specimens and patient information

Pancreas specimens were selected from seven non-diabetic patients undergoing full or partial pancreatectomy for concern about pancreatic cancer but in whom pathology demonstrated no malignant lesions involving pancreas itself or evidence of pancreatitis. At the time of surgery pieces of tissue were placed in Tissue-Tek OCT (Sakura Finetek U.S.A., Inc., Torrance, CA), frozen in chilled isopentane and stored at −80°C pending sectioning. Medical records were reviewed for the presence or absence of diabetes, complications of diabetes, treatment of diabetes or concomitant medications known to directly alter insulin secretion or sensitivity. Other parameters included: age, height, weight, history of glycemia including glucoses, glycohemoglobin levels, and pancreas pathology diagnosis. Via review of both the Partner's Healthcare Research Patient Data Repository and confirmatory review of anatomic pathology information system records present in the Harvard/Partners Virtual Specimen Locator, it was determined that none of the selected patients had a history of diabetes or anti-diabetic drug use, and all determined pre-surgical blood glucose levels were normal. The clinical characteristics of the surgical patients are shown in Table 1.

**Table 1:** Clinical characteristics of 7 surgical patients.

Gender	Age	BMI	Blood Glucose (mg/dL)	Diagnosis
M	81	23	92	Duodenal adenocarcinoma with metastasis to lymph nodes, but not pancreas.
M	60	24	85	Stage IIA bile duct carcinoma with focal infiltration of pancreas
F	67	26	Unknown	Pancreatic mucinous cystadenoma
M	72	35	92	Invasive periampullary adenocarcinoma No pancreatic involvement.
M	72	22	81	Periampullary tubulovillous adenoma with focal high grade dysplasia.
F	57	24	90	Intraductal papillary pancreatic mucinous cyst without malignancy.
F	77	26	91	Intraductal papillary mucinous neoplasm

### Transplanted Islets

Pancreases from the New England Organ Bank were processed in the Joslin Islet Cell Resource Center. Donor HO5-25 was a 35 year-old male with a gunshot wound to the head. He had no history of diabetes, a BMI of 30.4, was in intensive care for three days, and recorded glucoses ranged between 106–175 mg/dl. Cold ischemia time for the pancreas was 5 hours. Donor HO6-16 was a 57 year-old male with a broken neck from a fall. He had no history of diabetes, a BMI of 27.7, was in intensive care for 1.5 days and recorded glucoses ranged from 133–160. Cold ischemia time for the pancreas was 6 hours. Islets were isolated using standard techniques [17] from two cadaver donors and 500 islets were transplanted under the kidney capsule of 6 non-diabetic ICR-SCID mice. In both cases, islet purity was 80–90% and viability was 90–95%. Blood glucose concentrations were determined using a glucose meter (Precision QID; Abbott Labs, Bedford, MA) with blood obtained from a snipped tail.

After 28 days, the mice were anesthetized, the islet grafts were recovered and placed in cryomolds, embedded in Tissue-Tek OCT (Sakura Finetek U.S.A., Inc., Torrance, CA), frozen in chilled isopentane and stored at −80°C, pending sectioning at 8µm;the mice were then sacrificed.

### Laser Capture Microdissection (LCM)

LCM was performed using a protocol modified for human pancreatic tissue [18], [19]. For the surgical specimens, frozen pancreatic sections were dehydrated in 70% ethanol for 30 seconds, DEPC-treated water for 30 sec, 70% ethanol for 30 sec, 100% ethanol (rinsing), 100% ethanol twice for 1 min, and xylene for 4 min. Immediately after the slides were completely air-dried, LCM was performed using PixCell II Laser Capture Microdissection System (Arcturus Engineering, Mountain View, CA). LCM was performed under direct microscopic visualization by melting thermoplastic films mounted on optically transparent LCM caps (Arcturus) on selected populations of cells; β-cell rich tissue was identified by intrinsic fluorescence [18]. Care was taken to not dissect tissue from any areas with abnormal histological appearance. The laser power and pulse duration determined the laser spot size. To obtain optimal size of the laser pulse, we set the system parameters as follows: laser power, 35 mW; pulse duration, 3.0 msec; and spot size, 7.5 µm. The cells attached to the thermoplastic transfer film were incubated with 10 µl of a guanidine thiocyanate and polyethylene glycol octylphenol ether-based buffer for 30 min at 42°C. Each section typically had 3–10 islets, and each islet contained 2–4 clumps of intrinsic fluorescence representing β-cells. On average, 6–7 sections were used to obtain 800 hits, which were needed to obtain sufficient RNA for the array. This minimum laser spot size of 7.5 µm allows collection of tissue from only a few cells. LCM on the transplanted islet tissue grafts was performed in an identical manner except the pulse duration was 2.5 msec and the thermoplastic film contained about 500 hits of the microdissected cells. For both pancreatic and graft dissections, each LCM session was completed within 30 minutes to avoid RNA degradation.

### RNA extraction, amplification biotinylation and GeneChip processing

Total RNA was isolated using PicoPure RNA Isolation Kit (Arcturus) and amplified by T7-based linear amplification using T7-oligo-dT-primers. RNA amplifications were performed using RiboAmp HS RNA Amplification Kits (Arcturus) following the manufacture's protocol. Amplified RNA (aRNA) quantity was evaluated spectro-photometrically by readings at 260 nm (A260) and 280 nm (A280). RNA quality was assessed by running 100 ng of aRNA on Nano LabChip of Agilent 2100 Bioanalyzer (Agilent Technologies, Inc., Santa Clara, CA). Amplified RNA was converted into double-stranded complementary DNA (cDNA) using the RiboAmp HS RNA Amplification Kit (Arcturus), and biotinylated complementary RNA (cRNA) was generated from cDNA by *in vitro* transcription reaction using the BioArray High Yield RNA Transcript Labeling Kit (Enzo Diagnostics, Farmingdale, NY). RNA products were purified using the MiraCol™ Purification Columns (Arcturus). Biotinylated cRNA was fragmented to nucleotide stretches of 30–200 nucleotides and hybridized to the GeneChip Human X3P Array (Affymetrix, Santa Clara, CA) after the quality of the labeled cRNA was checked by GeneChip test array. The GeneChip X3P array contains 61,000 probe sets representing 47,000 transcripts and variants, including approximately 38,500 well-characterized human genes. The probe arrays were washed and stained using the Fluidics Station 400 and scanned using the Affymetrix Gene Chip Scanner 3000 (Gene Chip Expression Analysis *Technical Manual*, Affymetrix). Microarray experiments were run at the Genomics Core of the Joslin Diabetes Center.

### Microarray data analysis

Array data were normalized and comparisons were performed using the DNA-Chip Analyzer (dChip) software (Harvard School of Public Health, Boston, MA). dChip software implements invariant set normalization and probe-level model-based expression analysis on multiple arrays, and computes the t-statistic and the p-value based on the t-distribution. Computation of standard errors for expression indexes allows calculating confidence intervals for fold changes (33,34). Lower confidence bound (LCB) and p-value were used to assess differentially expressed genes using the cutoff 1.2 and p<0.05, respectively. All data will be deposited in a MIAME compliant data base - accession number pending.

### Data analysis

Results are expressed as mean ± standard error (SE).

## Results

### Blood glucose levels of mice

Glucose levels of mice with islet transplants ranged from 133 mg/dL to 178 mg/dL through 4 weeks. These values are high for humans – in the impaired glucose tolerance range. The actual levels in these mice may be even a little higher because clinical glucose test strips give artifactually low values in rodents [20]. Even these mild elevations can cause glucotoxicity [6]. However, blood glucose levels in this range approximate the mildly hyperglycemic environment of islets in successful human islet transplants [16]. Blood glucose data for recipient mice is shown in Table 2.

**Table 2:** Non-Fasting Blood Glucose of Recipient ICR-SCID Mice.

Graft ID	Donor ID	No. of IEQ	Blood Glucose (mg/dl)		
		IEQ	0 D	14 D	28 D
1	**H05-25**	500	138	147	141
2	**H05-25**	500	169	177	163
3	**H05-25**	500	161	151	178
4	**H06-16**	500	142	158	114
5	**H06-16**	500	153	133	160
6	**H06-16**	500	139	142	126
		Mean	**150**	**151**	**147**

Graft IDs represent separate transplants of 500 IE each. Donor ID refers to two separate cadaver donors (H05-25 and H06-16). IEQ refers to islet equivalents. Blood glucose values were determined on day 0 (pre-transplant), 14 and 28 after the transplants.

### IRE-1 Pathway

IRE-1 splices transcription factor XBP-1 mRNA to its active form, resulting in additional translation of transcription factor XBP-1, which increases expression of genes for ER chaperone proteins and ER degradation proteins [7]. This pathway likely serves a protective function for beta cells, helping to increase the cell's protein-folding capacity and ensuring quality control by providing additional tools for degrading misfolded proteins [21]. A number of the downstream targets of this pathway were significantly upregulated in transplanted beta cells as compared to surgical controls. Data for the IRE-1 pathway are contained in Table 3. Upregulated gene expression of the following targets was found: XBP-1; the chaperone proteins PDIA4 (protein disulfide isomerase family A, member 4), Bip, and Grp94 (HSP90B1, heat shock protein 90kDa beta); and the ER degradation proteins (EDEM1, EDEM2). Some other genes involved with the ERAD machinery [22], DERL1 (Der1-like domain family, member 1) and DERL3, were down regulated. ERdj4 (DNAJB9, DnaJ (Hsp40) homolog, subfamily B, member 9), which can inhibit ER stress-induced apoptosis perhaps by aiding chaperone function [23], was upregulated. DNA-JC3 was also upregulated. There was no differential expression of WFS1, which is another IRE-1 target [24]. Interestingly, JNK (c-jun N-terminal kinase), which is activated by both the IRE-1, and XBP1 pathways [25] and has been implicated in beta cell apoptosis and dysfunction [26], had down regulated expression.

**Table 3:** IRE-1 Pathway.

		Control		Transplant				
Probe ID	Gene Name	Value	SE	Value	SE	Fold Change	LCB	p-value
**Hs.129166.0.A1_3p_at**	IRE-1	11	3	10	1	−1.09	−0.59	0.7862
**g4827057_3p_s_at**	XBP1	4825	221	6970	261	1.44	1.3	0.0002
**g7662001_3p_at**	EDEM1	317	34	205	27	−1.54	−1.17	0.0280
**g8922666_3p_at**	EDEM2	126	4	176	15	1.39	1.16	0.0294
**g13623480_3p_s_at**	PDIA4	212	35	1167	147	5.51	3.93	0.0010
**Hs.75410.1.S1_3p_at**	BiP	644	41	1445	331	2.25	1.37	0.0617
**g4507676_3p_a_at**	Grp94	3228	166	4347	309	1.35	1.14	0.0223
**Hs2.429981.1.A1_3p_s_at**	DNAJC3	18	4	135	20	7.56	4.63	0.0016
**Hs.267445.0.S2_3p_at**	JNK	181	19	73	18	−2.48	−1.67	0.002
**g13376995_3p_at**	WFS1	210	19	203	20	−1.03	−0.81	0.835
**g5262493_3p_a_at**	ERdj4	2464	209	4136	460	1.68	1.31	0.015
**Hs.241576.0.S2_3p_at**	DERL1	899	49	598	101	−1.5	−1.15	0.032
**Hs.14587.2.A1_3p_at**	DERL3	108	7	73	5	−1.49	−1.24	0.004

### PERK Pathway

PERK is activated by decreasing levels of free BiP in the ER, and is inhibited by DNAJC3 which acts to turn off this portion of the ER stress response. PERK initiates two signal cascades. In the first pathway, PERK phosphorylates eIF2α resulting in generalized attenuation of translation but with selectively increased translation of transcription factor ATF-4. The transcriptional cascade initiated by ATF-4 leads to transcription of ER chaperone proteins (BiP), ER degradation associated gene products (HERP1, HERP2), pro-apoptotic associated gene products such as C/EBP homologous protein (CHOP, DDIT3) and proteins like GADD34 (PPP1R15A), which participate in feedback inhibition of the cascade. This pathway also leads to transcriptional inhibition of gene products involved in glucose metabolism (FBP, PEPCK, IRS-2). In the second pathway, PERK activates NRF2 (nuclear factor [erythroid-derived 2]-like 2), which turns on an antioxidant response element signaling pathway [27], thus leading to transcription of antioxidant gene products: glutathione S-transferase alpha 1(GSTA1), heme oxygenase-1 (HMOX1), Thioredoxin (TXN), NAD(P)H dehydrogenase, quinone 1 (NQO1) and, glutamate-cysteine ligase (GCLM) [7], [14].

The gene expression data of the PERK pathway are contained in Table 4. Both signal cascades initiated by PERK were downregulated in the transplanted islets as compared to controls. Both of PERK's direct substrates, EIF2A and NRF2, showed markedly reduced expression in transplanted islets. EIF2A downregulation could be protective by inhibiting the production of proteins that have folding problems. Two factors are activated by stress to dephosphorylate EIF2A and attenuate protein synthesis: GADD34 (PPP1R15A) and CReP (PPP1R15B) [28]. Expression of GADD34 was unchanged in the transplanted beta cells, but that of CReP was markedly downregulated, which could compensate for the reduction in EIF2A. The reduction in NRF2 could pose a threat through reduced expression of the antioxidant genes GCLM, NQO1 and TXN. Expression levels of GSTA1 and HMOX were unchanged.

**Table 4:** PERK Pathway Dataset.

		Control		Transplant				
Probe ID	Gene Name	Value	SE	Value	SE	Fold Change	LCB	p-value
**g11125767_3p_a_at**	PERK	1912	160	1956	162	1.02	0.83	0.8574
**g13182760_3p_at**	EIF2A	1217	126	288	61	−4.23	−2.9	0.0002
**g4502264_3p_at**	ATF4	3609	215	3842	241	1.06	0.9	0.5411
**g4502262_3p_s_at**	ATF3	18	6	34	6	1.88	1.03	0.1099
**g13177717_3p_at**	CHOP	348	43	159	26	−2.19	−1.57	0.0041
**4901426C_3p_s_at**	GADD34	97	11	107	12	1.1	0.84	0.5526
**Hs.17448.0.S1_3p_at**	CReP	509	46	220	44	−2.31	−1.66	0.0009
**Hs.75410.1.S1_3p_at**	BiP	644	41	1445	331	2.25	1.37	0.0617
**Hs2.429981.1.A1_3p_s_at**	DNAJC3	18	4	135	20	7.56	4.63	0.0016
**g439225_3p_at**	FBP1	45	6	55	5	1.22	0.9	0.3094
**g4511968_3p_a_at**	IRS2	1488	165	814	122	−1.83	−1.35	0.0085
**g4505638_3p_a_at**	PCK1	172	53	2204	567	12.83	6.43	0.0164
**Hs.146393.1.S1_3p_a_at**	HERP1	2783	117	2706	428	−1.03	−0.81	0.8689
**Hs.30211.0.S2_3p_at**	HERP2	629	81	337	75	−1.87	−1.25	0.0250
**g5453775_3p_at**	NRF2	699	60	259	44	−2.7	−1.99	0.0002
**g4504170_3p_at**	GSTA1	16	7	105	40	6.74	2.14	0.0800
**g4504436_3p_at**	HMOX	20	2	25	3	1.22	0.9	0.2951
**g4505414_3p_a_at**	NQO1	215	21	131	5	−1.64	−1.32	0.0101
**Hs.315562.0.A1_3p_at**	GCLM	229	30	118	21	−1.94	−1.34	0.0147
**g11345419_3p_s_at**	Thioredoxin	455	27	184	21	−2.48	−2	0.0000
**g5813798_3p_at**	AATF	572	49	434	90	−1.32	−0.94	0.218
**g13376995_3p_at**	WFS1	210	19	203	20	−1.03	−0.81	0.835
**g11056039_3p_at**	trb3	382	58	538	121	1.41	0.84	0.285
**g4502258_3p_a_at**	ASNS	828	51	576	107	−1.44	−1.06	0.081
**Hs.99029.0.S2_3p_at**	C/EBP-beta	103	8	46	4	−2.23	−1.83	0.000
**Hs.7887.0.A1_3p_at**	C/EBP-gamma	80	10	50	10	−1.6	−1.1	0.064
**g7657068_3p_a_at**	ERO1	139	13	155	17	1.12	0.86	0.495

Other downregulated genes included HERP2 (HEY2), insulin receptor substrate 2 (IRS-2), CHOP and C/EBP-beta. Activation of IRS-2 appears to have an antiapoptotic effect on beta cells [29]. However, the downregulation of CHOP, a key inducer of ER stress induced apoptosis [14] and that of JNK suggest that ER stress in this circumstance may not be detrimental to the survival of transplanted beta cells. Moreover, C/EBP-beta, which is also potentially proapoptotic, was also downregulated [30].

Various other genes thought to be targets of the PERK pathway had no significant change of expression. These include the following antiapoptotic genes: AATF (antiapoptosis-inducing transcription factor) [31] and WFS1 (Wolfram syndrome 1) [24]. Genes implicated as being proapoptotic include: TRB3 (tribbles homolog 3), which is downstream of CHOP [32] and ERO1 (ER oxidase 1 alpha), which may exert proapoptotic effects by stimulating inositol 1, 4, 5-triphophate receptors [33]. Another gene with unchanged expression was ASNS (asparagine synthetase), which can be activated by isoforms of AFT-3 [34]. A very interesting change was an enormous increase in PCK1 (phosphoenolpyruvate carboxykinase 1) in the transplanted beta cells. While PCK1, a key enzyme for gluconeogenesis, has been linked to ER stress, it is also known to be almost absent in beta cells, which have little if any gluconeogenesis [35]. This change fits with the pattern of phenotypic change seen with glucotoxicity [36].

Thus, the PERK arm of the ER stress response appears to be mostly downregulated in transplanted islets. It is possible this is in part due to increased expression of the PERK inhibitor DNAJC3 [15], which can be activated by both the IRE-1 and ATF6 pathways.

### ATF-6 Pathway

As free levels of BiP decrease in the ER, ATF-6 translocates to the Golgi apparatus where the active transcription factor component, ATF-6a, is cleaved and released. ATF-6a then translocates back to the nucleus where it works with cofactor NFY (nuclear factor Y) to induce transcription of ER-associated degradation proteins (HERP1, HERP2, ERO1), ER chaperone proteins (BiP), pro-apoptotic gene products (CHOP), and PERK inhibitor DNA-JC3 [7]. The data related to the ATF-6 pathway are shown in Table 5. There was significantly decreased expression of ATF-6 in the transplanted beta cells, as well as downstream gene products CHOP and HERP2 (HEY1), which can be activated by JNK. There was increased expression of HRD1 (SYVN1, synovial apoptosis inhibitor 1), which exerts an antiapoptotic effect by degrading unfolded proteins [37]. Paradoxically, there was increased expression of DNAJC3. The ATF-6 pathway serves to protect the cell by increasing folding capacity as well as to induce apoptosis if the folding capacity remains overwhelmed. It appears that this pathway is down regulated when beta cells are in a hyperglycemic environment.

**Table 5:** ATF-6 Pathway Dataset.

		Control		Transplant				
Probe ID	Gene Name	Value	SE	Value	SE	Fold Change	LCB	p-value
**Hs.40328.0.A1_3p_at**	ATF6	309	15	207	26	−1.49	−1.19	0.0145
**Hs.797.1.S3_3p_at**	NFY	94	22	52	6	−1.81	−1.08	0.1082
**g13177717_3p_at**	CHOP	348	43	159	26	−2.19	−1.57	0.0041
**Hs2.429981.1.A1_3p_s_at**	DNAJC3	18	4	135	20	7.56	4.63	0.0016
**Hs.146393.1.S1_3p_a_at**	HERP1	2783	117	2706	428	−1.03	−0.81	0.8689
**Hs.30211.0.S2_3p_at**	HERP2	629	81	337	75	−1.87	−1.25	0.0250
**Hs.108689.0.A2_3p_at**	SREB2	16	2	16	4	−1.02	−0.61	0.9535
**Hs.75410.1.S1_3p_at**	BiP	644	41	1445	331	2.25	1.37	0.0617
**Hs.25740.0.S1_3p_a_at**	ERO1	169	21	169	27	1	−0.7	0.9992
**Hs.75859.1.A1_3p_at**	HRD1	105	6	183	24	1.75	1.33	0.024

## Discussion

The present study provides unique information about ER stress in human beta cells exposed to hyperglycemia in an *in vivo* situation. LCM was used on frozen sections to obtain beta cell rich tissue. To avoid the potential artifacts of cadaver pancreases due to premorbid illness and cold ischemia time, fresh tissue was obtained from pancreases of non-diabetic subjects undergoing surgery. Gene expression of these beta cells in the pancreas could then be compared with human beta cells that were transplanted into mice. An important point is that glucose levels in mice are naturally higher than truly normal levels in humans; they are in the range of impaired glucose tolerance or mild diabetes. Thus, these beta cells are in a metabolic milieu similar to those in a liver site of patients with successful islet transplants, who almost always have impaired glucose tolerance rather than truly normal glucose levels [16]. We know that beta cell function is adversely affected by glucose toxicity even with these mild glucose elevations [6]. Thus, these comparisons make it possible to obtain insights into the ER stress response in a transplant situation and with exposure to glucose toxicity. However, from these experiments it is not possible to be certain about how much differential expression is due to glucotoxicity versus that from the abnormal environment of a graft site. There are certainly factors other than mild hyperglycemia that could have accounted for these gene expression changes. Beta cells in a graft site have reduced vascularization, exposure to relative hypoxia and altered topographical relationships between beta and not beta cells [38], [39]. There could even be a variety of other perturbations such as exposure of human cells to mouse serum.

There is great interest in the contributions of ER stress in beta cells to the pathogenesis of diabetes and the failure of transplanted human islets to maintain insulin secretion. It is important to recognize the protective versus the destructive aspects for ER stress. Much of the unfolded protein response (UPR) is protective in that chaperones are induced, unfolded proteins are degraded and protein synthesis is reduced. The IRE-1 pathway is particularly important for this protective response [21]. However, when ER stress becomes severe, apoptosis pathways can be induced, with CHOP and JNK playing important roles. The most striking thing about the present study is that beta cells in a transplant site exhibit UPR with changes that should help the cells adapt to unfolded proteins and to resist apoptosis. Of the three pathways of ER stress, the IRE-1 pathway seems the most activated; the other pathways have some variable results but seem mostly downregulated. It is especially noteworthy that two factors well known to be associated with apoptosis, CHOP and JNK, are downregulated.

These findings raise important questions about the relationship between ER stress and beta cell death in diabetes. Beta cell death elicited by cytokines and amyloid has been dissociated from ER stress [40], [41], but free fatty acid (FFA)-induced cell death *in vitro* has been linked [42]. However, deleterious effects of FFA on beta cells in diabetes remain to be established [6]. Beta cells in the present study exposed to mild hyperglycemia have a clear UPR response, which appears to be adaptive and protective against excessive accumulation of unfolded proteins. It seems likely that most beta cells have this UPR response and the only a small minority of vulnerable cells die from the proapoptotic mechanisms of ER stress. This hypothesis fits well with the finding that in pancreases of subjects with type 2 diabetes, only rare beta cells are stained for CHOP [12]. In summary, this study shows that human beta cells in a transplant site have many changes in the expression of ER stress genes. The most dominant changes are adaptive and protective and not proapoptotic.
